# Health financing reform in Uganda: How equitable is the proposed National Health Insurance scheme?

**DOI:** 10.1186/1475-9276-9-23

**Published:** 2010-10-13

**Authors:** Juliet Nabyonga Orem, Charlotte Muheki Zikusooka

**Affiliations:** 1 Health systems and Services Cluster, WHO Uganda Country office, Kampala, Uganda; 2 HealthNet Consult, Kampala, Uganda

## Abstract

**Background:**

Uganda is proposing introduction of the National Health Insurance scheme (NHIS) in a phased manner with the view to obtaining additional funding for the health sector and promoting financial risk protection. In this paper, we have assessed the proposed NHIS from an equity perspective, exploring the extent to which NHIS would improve existing disparities in the health sector.

**Methods:**

We reviewed the proposed design and other relevant documents that enhanced our understanding of contextual issues. We used the Kutzin and fair financing frameworks to critically assess the impact of NHIS on overall equity in financing in Uganda.

**Results:**

The introduction of NHIS is being proposed against the backdrop of inequalities in the distribution of health system inputs between rural and urban areas, different levels of care and geographic areas. In this assessment, we find that gradual implementation of NHIS will result in low coverage initially, which might pose a challenge for effective management of the scheme. The process for accreditation of service providers during the first phase is not explicit on how it will ensure that a two-tier service provision arrangement does not emerge to cater for different types of patients. If the proposed fee-for-service mechanism of reimbursing providers is pursued, utilisation patterns will determine how resources are allocated. This implies that equity in resource allocation will be determined by the distribution of accredited providers, and checks put in place to prohibit frivolous use. The current design does not explicitly mention how these two issues will be tackled. Lastly, there is no clarity on how the NHIS will fit into, and integrate within existing financing mechanisms.

**Conclusion:**

Under the current NHIS design, the initial low coverage in the first years will inhibit optimal achievement of the important equity characteristics of pooling, cross-subsidisation and financial protection. Depending on the distribution of accredited providers and utilisation patterns, the NHIS could worsen existing disparities in access to services, given the fee-for-service reimbursement mechanisms currently proposed. Lastly, if equity in financing and resource allocation are not explicit objectives of the NHIS, it might inadvertently worsen the existing disparities in service provision.

## Introduction

Equity in health has been defined as the absence of systematic disparities in health between social groups who have different levels of underlying social advantage/disadvantage [1]. Inequalities in health are an effect of differential distribution of several determinants of health, including health care. Equity in health care financing is assessed by the degree of inequality in paying for health care between household of unequal ability to pay [2]. The purpose of health financing is to make funding available, as well as to set the right financial incentives for providers, to ensure that all individuals have access to effective public health and personal health care. To ensure that individuals have access to health services, three interrelated functions of health system financing are crucial: revenue collection, pooling of resources, and purchasing of interventions. Categories of health care financing sources include, taxation, donor funds, social health insurance, private health insurance, other private sources like NGOs own resources and out-of-pocket (OOP) expenditures. Reliance on private OOP spending remains significant, constituting over 40% of total health expenditure in 31 countries in sub-Saharan Africa (SSA) [3]. The poor spend disproportionately more than the rich as a percentage of household income on health care [4], thus making OOP a very regressive form of financing. OOP expenditure on health has been noted to be highly inequitable and predisposes households to incurring catastrophic expenditures and impoverishment [5]. In order for a financing mechanism to be equitable, collection of contributions should be progressive, implying that people with higher income, pay progressively higher proportions of their income and vice versa. Therefore, progressive health financing dictates that the financing burden borne by poorer income groups is less than that borne by higher income groups. Equity is embedded in the principles of social justice which dictate that access to health care is every citizen's right, and ought not to be influenced by income and wealth. Health care should be distributed according to need and financed according to ability to pay [6].

In 2005, WHO member states adopted a resolution encouraging countries to develop sustainable and equitable health financing systems capable of achieving universal coverage [7]. In 2008, WHO defined universal coverage as "securing access for all to appropriate promotive, preventive, curative and rehabilitative services at an affordable cost" Most developing countries have undertaken health financing reforms where, among the guiding principles equity is envisioned to be addressed. Like many other countries, Uganda is currently proposing to start a National Health Insurance (NHI) scheme. Within this insurance scheme, addressing equity concerns is central to the design process and the scheme aims to increase welfare gain in health care through financial risk protection. It is hoped that the NHI would pool resources from private sources, thereby promoting equity through cross-subsidies. The extent to which the NHI will achieve equity objectively will largely be depended on its design. In this paper, we analyse the extent to which the proposed National Health Insurance Scheme (NHIS) will address equity concerns within the overall health financing landscape in Uganda.

In this paper, health equity is interpreted as fairness in financial contribution towards health care and fairness in benefiting from health services. Equitable health financing refers to a system that promotes making financial contributions in relation to people's ability to pay (with the poor paying less and the rich more) while offering access to health services according to their need for care, and that services meet conditions of quality, opportunity, and dignity, regardless of people's ability to pay. This means that populations should have access to needed health interventions without the risk of financial catastrophe. Preferably, funds collected should cover the risks of all people, and should provide cross subsidies between rich and poor people.

## Background

### Organisation of the health care delivery system

Uganda's health care delivery system is organized at several levels. The National referral hospitals provide comprehensive specialist services and, in addition, they are involved in teaching and research. Regional referral hospitals provide general curative and preventive services, specialist services, in-service training and research. A general hospital provides general curative and preventive services, in-service training, consultation and research to community based health care programmes. A Health Centre (HC) IV provides general preventive and curative services, emergency surgery and blood transfusion services. HC III and II, which are categorized lower level health units/facilities, provide mainly ambulatory services as included in the Uganda Minimum Health Care Package of services. HC IIIs also provide laboratory services for diagnosis, maternity care and first referral cover for HC II in their areas of jurisdiction. The HC IIs only provide out patient care and community outreach services; hence they provide the first level of interaction between the community and formal health services. Functionality of these health facilities has been noted to be sub optimal with lower levels of care affected more than higher levels. For example, percentage of HC IIs experiencing stock outs of essential medicines has been close 80% between 2006 to 2009; the national average stands at close to 70% [8]. Only 52% of HC II were able to provide antenatal care compared to hospitals at 95% [9]. Percentage of HCIIs, offering child immunization with all equipment available, is only 55% compared to hospitals at 90% [9].

It is difficult to assess the NHIS without understanding the context within which it would be implemented.

### Context within which NHIS would be implemented

Uganda's estimated GDP per capita is US$320 and 31% of the population live below the poverty line [10]. Inequalities exist between rural and urban areas and the different regions of the country. Thirty four percent (34%) of the population in rural areas live below the poverty line compared to 14% in urban areas, while 61% of the population in Northern Uganda lives below the poverty line compared to 16% in the central region [10].

#### Human resources

Currently, the proportion of approved posts filled by trained health professionals is 51% [11]. Using payroll information, the proportion of districts with agreed minimum positions filled by trained health professionals is 15% [12]. Minimum staffing level implies a staffing level of 80% of set norms. There are inequalities in the distribution of human resources for health by region, districts, rural and urban areas and level of care. Highly trained cadres like medical doctors, degrees and specialised nurses/midwives, pharmacists, dentists as well as diagnostic personnel are extremely unequally distributed, serving only a small fraction of the population, largely in urban areas. Although about 80% of the Ugandan population live in rural areas, the urbanised central region that houses only 27% of the population has 64% of qualified nurses and midwives, 71% of medical doctors, 76% of dentists and 81% of pharmacists [11]. Due to salary disparities between the public and Private not for profit (PNFP) sectors, attrition of health workers from the latter to the former has been documented [8].

#### Access to health facilities

The percentage of the population residing in 5 km of a health facility (public/Private not for profit (PNFP) is 48% (2004/5). There are variations in access to the different levels of health facilities; only 11% of the population lived within a 5 km radius of a hospital, 23% for health centres and 49% for private clinics [10].

#### Infrastructure

Distribution of infrastructure is highly inequitable. The national average is 8,785 population per facility. This varies from 1:20,376 in some rural districts to 1:5,295 in Kampala, the capital city [13]. Distribution of hospitals is also inequitable with sixteen out of 80 districts having no hospital, while several districts have more than two hospitals with an extreme for Kampala having eight hospitals. The level of functionality of the different facilities also varies considerably; functionality of available equipment is as low as 33% at the general hospital level, 52% at the Health Centre four (HC IV) level and 44% at the regional referral level [14].

#### Financing

In Uganda, households constitute a major source of health financing (49%) followed by donors (35%) and then government (15%). NGOs contribute less than 1% [15]. User fees were abolished in all government health facilities in March 2001, but at hospital level, a dual system exists. There is a free wing for those who cannot pay and a paying wing for those who can afford. The private sector charges user fees and there is evidence of patients paying under-the counter fees in public institutions [16].

The health sector in Uganda has been faced with a challenge of under-funding for a very long time. GoU expenditure on health remained low at around US$5 -7 per capita between 2004/05 to 2007/08 [17]. The projected allocation for health for the period 2010/2011 to 2012/2013, shows no increase in the health sector budget [18]. Donor funding through projects amounted to US$10 per capita in 2008/09 [17]. This is not adequate to deliver a minimum health care package costed at US$40 per capita [19]. Gaps in service delivery are evident with only 35% of health facilities having essential medicines throughout the year and only 51% of approved posts filled by trained health workers [12].

Out of pocket expenditure for health continues to increase amidst government provision of free health services. Households spend about 9% of their household consumption expenditure on health [20]. Average cost per utilization, computed as the average cost per person for those who fell ill and sought care, increased from US$7 per utilization in 2003 to US$13 in 2005 [20]. Nearly 50 percent of total household health expenditure is incurred on drugs. Twenty eight percent (28%) of the households in Uganda are experiencing catastrophic payments, that is health expenditures in excess of 10 percent of total household consumption; with considerable variations by wealth quintile (28.3% among the poorest quintile -24.8% among the richest quintile) and region (23.6% central region to 38.1% western region). Percentage of households incurring catastrophic health expenditure increased from 8% to 28% between 1996 and 2006, despite the elimination of user fees in 2001 [20]. Two percent of households (2.3%) were pushed into impoverishment because of medical bills [20]. Private health insurance, which is largely subsidized by employers on behalf of employees, is for a few covering only 0.2% of the population [15].

Currently, government resources are allocated to decentralized sub units (districts and hospitals) using a resources allocation formula. The formula incorporates variables like, population, child mortality as a proxy for health need, district topography as a proxy for cost of service delivery and poverty index of the district as a proxy for deprivation. In the case of hospitals the formula uses bed capacity. Inequitable allocation of government funding has been documented, conflict affected districts, with high poverty indices and worse health indicators received less funding than better off districts [21]. Local governments (districts) are autonomous entities that collect revenue that can be used to provide health services. This however remains very low contributing on average 2% of revenue.

## Methodology

This study is a qualitative assessment of a reform process. In the assessment, we reviewed policy documents, budget framework papers and the medium term expenditure framework. In addition, we reviewed government documents to assess the level and distribution of resources like infrastructure, human resource and funding for the health sector. We also relied on the experiences obtained during our participation in National Task Force on Insurance (that meets monthly since 2007) and stakeholder consultative meetings. Discussions and consultation on designing the NHIS have been ongoing since 2006. Lastly, we relied on our current knowledge of Uganda's overall financing landscape, of which the proposed NHIS would be a part.

In assessing whether the proposed NHIS is equitable, we considered the key principles of equity in health financing. Using the Kutzin framework [22], we critically assessed the proposed health insurance scheme, focusing on the following aspects: (a) collection of funds; (b) pooling of resources and proportions of the population to be covered by the scheme; (c) purchasing of services; and (d) provision of services and the benefit package available to those who are covered. This analysis takes into consideration the potential impact of the proposed NHIS on the existing financing mechanisms, and the impact this will have on access to services and on provision of services, in both the public and private sectors. In addition, the analysis framework for this assessment is based on the basic principles of fair financing. Equitable financing is based on set of principles, namely: *financial protection *(no one in need of health services should be denied access due to inability to pay and, households' livelihoods should not be threatened by the costs of health care); *progressive financing *(contributions should be distributed according to ability-to-pay, and those with greater ability-to-pay should contribute a higher proportion of their income than those with lower incomes); and *cross-subsidies *(from the healthy to the ill and from the wealthy to the poor).

Taking into consideration these frameworks, we critically reviewed the current NHIS design and the revenue and expenditure simulation results, proposed enrolment schedules and contribution rates. The revenue and expenditure analysis results derived using the health insurance simulation model (SIMNS) have been agreed as the working position by the National Task Force on Health Insurance.

## Results

### Overview of the National Health Insurance Scheme (NHIS)

The development of the NHIS is hinged on the WHO resolution [7], Uganda's Constitution [23], the National Health Policy [24] and the Health Sector Strategic Plan I & II [25]. The 1995 Constitution emphasises that the state shall take all practical measures to ensure the provision of basic medical services to the population. The goal of the National Health Policy (NHP) is to ensure attainment of a good standard of health by all Ugandans and to promote a healthy and productive life [24].

The Health Sector Strategic Plan I (2000/1-2004/5) and II (2005/6-2009/10) highlighted development of alternative health financing mechanisms among which is Social health insurance (SHI) [25]. A feasibility study undertaken in 2001 recommended that Uganda pursues a strategy of starting up SHI gradually, by initially covering only civil servants and their families located in large cities plus workers and their families employed by large companies such as those employing 250 workers or more. The study recommended that step-by-step, the scheme could be expanded to include all workers and their families in the formal sector, and hopefully the informal sector as well [26]. In 2006, the Government of Uganda asked the MoH to design a Health insurance (HI) scheme through a cabinet minute No. 63 (CT 2006) to the Ministry of health (MoH). The Minister of Health established a national task force on health insurance, with representation from all relevant stakeholders to spearhead drafting of the Bill and design issues. Stakeholders include MoH, Ministry of Finance, Ministry of labor and gender, Ministry of public service, trade Unions, Federation of Uganda employers. The purpose of the Bill is to diversify and strengthen health care financing, stimulate providers to provide good quality services at affordable prices and increase welfare gain in health care through financial risk protection.

### Collection of funds

The scheme design is at an advanced stage and revenue and expenditure simulation has been undertaken. The scheme proposes to enroll all public servants initially, three years later enrol formal private sector over a period of three years, and enrolment of the informal sector starting in year seven gradually attaining universal coverage in 15 year's time. In the interim, the formal private sector employees have an option of making their own arrangements with private insurance providers on a voluntary basis. Revenue and expenditure simulation has estimated a 4% payment from salary from the formal sector employee (public and private sector) with an additional 4% from the employer. In the case of civil servants, the 4% will be paid by the government. It is estimated that 25% of the population are categorised the poorest of the poor [10], these will join the scheme from year seven on wards and their premium will be paid for using subsidies from either government or donor funds. Each paying member can bring along four (4) dependants to benefit from the scheme. According to the plan, before the informal sector and the poorest of the poor get onto the insurance scheme, free government services will be improved to ensure that they also access an acceptable quality of services. They may also enrol in community health insurance schemes on a voluntary basis.

In the current revenue and expenditure simulation, no copayments or deductibles are envisaged. Given the proposed contribution rates noted previously, the formal sector would be paying 4% of their salaries and employer paying 4%. Average annual wages for public employees are UgShs1,115,270 (US$558). The 4% represents annual contribution of UgShs44,612 (US$22) per paying member. With an additional 4% from the government (US$22) per government employee enrolled into the scheme. The informal sector will pay an annual contribution of UgShs 80,000 (US$41) per paying member. The challenge remains the fact that the informal sector which is non-taxable is large, accounting for close to 90% of the work force, and a significant proportion of these fall in the poorest quintile. These may be better reached through government proving services in full through tax revenues.

The proposal for the scheme to deduct a standard proportion of income (4%), rather than a flat fee, on one hand is considered to be progressive because people would make different contributions on the basis of their income levels. On the other hand, however, an even more progressive contribution would be that which charges a lower percentage contribution for lower incomes and a higher percentage for higher incomes. In view of this, the proposed contribution structure for the NHIS is only proportional but not progressive.

### Population covered and pooling of resources

It is proposed that the scheme starts with employees in the public sector. While this option is being considered in recognition of the fact that implementation needs to be gradual as experience in management of the scheme is developed, the strategy has equity and other important implications. Firstly, salaries in the public sector are relatively low compared to those in the private sector, implying that initially collections would be relatively low. Low collections are likely to impact on effectiveness of NHIS management and ability to provide a comprehensive benefit package of reasonable quality. Obviously, the smaller the amount of money collected, the smaller the package it can purchase. Average annual wages for public sector employees are UgShs1,115,270 (US$558). At a set contribution of 4% of employee salary and employer paying an additional 4%, this gives an annual contribution of US$44 per paying member plus the four (4) dependants translating into an annual contribution of US$9 per benefiting member. This is too low to buy a meaningful package of services. A minimum health care package in Uganda has been estimated to cost US$40 per capita [19]. Bringing the informal sector on board would pose even more challenges given the fact that the estimated contribution of US$41 per paying member is again low. A relatively bigger package would be possible with income subsidization in place, if the formal private sector is included (because of the higher salaries there). On the other hand, it is true that implementation of NHIS will be easiest starting with government employees, because the payroll is one place and the reform is relatively less likely to meet resistance as when it is proposed in the private sector.

Secondly, the speed at which the scheme is rolled out to other formally employed people in the private sector determines the extent to which the scheme is able to create a larger pool of funds and wider coverage. A smaller pool and low coverage will have less benefits of cross-subsidisation. Obviously, the performance of the scheme in the initial years (when only civil servants are covered) will highly determine the acceptability of the scheme by employees in the remaining formal sector.

Thirdly, the proposal to have NHIS covering employees in the public sector and later the employees in the remaining formal sector gradually may not result in a reasonable coverage for the existing population, mainly due to the high levels of unemployment and subsistence existence (especially in rural areas), as well as the existence of a growing informal sector that is not well regulated. This means that NHIS is likely to cover only a relatively small proportion of the population, and benefits of being covered by insurance will therefore be enjoyed by only a few people. The NHIS design proposes that for every contributing employee four dependents will be covered. Considering the large family sizes in Uganda and the fact that about 56% of the population are below the age of 18 years, this means that some dependents of contributing employees will not be covered by insurance. If one can make additional contributions per dependent, then can have more than four dependants enrolled on the scheme although details of how much is to paid for an additional dependants are yet to be worked out. In phase one, only 2.2% of the population will be covered and this will increase only minimally in phase 2 to 5 - 9% by the sixth year of implementing the scheme. Given this low coverage, the scope of NHIS to increase the scope for financial cross subsidisation would be limited.

### Purchasing of services

The proposed provider payment mechanisms include fee for service, capitation and a combination. The allocation of resources under the NHIS, if the option of paying providers on a fee-for-service basis is adopted, will be driven by the utilisation of services. The extent to which resource allocation is equitable (i.e. more resources being given to people or areas with the greatest need) depends on the degree to which frivolous use will be managed, and also on the extent to which access to services is equitable in the physical sense. In other words, if the NHIS accredits more providers in urban areas (e.g. because they have met the requirements of quality and scope of services available), it means that people living in the rural areas (who are covered by insurance) would have limited access to services, whether or not the need is greatest there. By implication, more resources would then end up in the places with the highest levels of utilisation, which might be partly due to better access to services. If these issues are not addressed, then resource allocation under NHIS might end up being inequitable, with a bias towards the places with the highest number of accredited providers.

### Provision of services and the benefit package

Gradual implementation also has implications for the arrangements with service providers. Providers for insured members will include public hospitals and Health Centre IVs (which are mini hospitals); accredited private-not-for-profit facilities and private-for-profit facilities. In the interim period when the scheme has been implemented only for employees in the public sector, there is likelihood for two-tier service provision where some services are available for people insured under NHIS and other people for instance, insured privately. This situation is likely to result in comparisons between benefit packages and quality of services for the different groups, and might negatively impact on acceptability of NHIS in the long-run.

The insured population under NHIS and those insured privately will access services from accredited providers. OOP payments will be made by those who seek care from the private sector while provision of free services will continue for the non insured seeking care in public facilities. Provision of free services in public facilities will continue to face challenges of inadequate inputs like, medicines stock-outs given the fact that projected allocation for health for the period 2010/2011 to 2012/2013, shows no increase in the health sector budget [18]. The implication of this modality, especially in the public facilities, would be provision of inferior quality services for the non insured compared to services provided to the insured population. On the other hand, continued payment of OOP especially in the private sector which is currently not regulated, impacts negatively on meeting financial risk protection objective.

### Integration with existing financing mechanisms

Uganda's sources of health care financing mainly include government, donors and households. There are some minimal contributions made through voluntary insurance and from firms. Currently, there is limited pre-paid financing, very limited financial protection (due to high out-of-pocket spending), fragmentation within and between financing mechanisms, and limited cross-subsidisation. Overall, the health care financing landscape is relatively inequitable and fragmented. One of the important potentials of the NHIS would be to address some of these challenges. Specifically, if successfully implemented, NHIS has the potential of improving cross-subsidisation through the creation of large pools (if high coverage is attained), reducing out-of-pocket spending, providing some financial protection, and reducing fragmentation between financing mechanisms. Unfortunately, the current NHIS design does not explicitly mention how the scheme would fit in with the existing financing mechanisms. So far, the scheme has been developed independently, without mention of how it would be integrated within the existing financing mechanisms. For example, there is no explicit mention of how publicly-funded services will be affected by the introduction of the NHIS (e.g. would the insurance contribution made by government for public employees be deducted from the total budget the health sector has been receiving? How will quality of services be ensured in the public facilities that are not accredited under NHIS? Should a person covered under the NHIS also be able to access the free services from public facilities?). Similarly, although the design mentions that private insurance will offer complimentary package, it is not clear what the role and collaboration arrangements of private health insurance agencies will be after the introduction of mandatory health insurance.

## Discussion

Earlier on, we noted that disparities exist in service provision in Uganda, with rural areas having far less inputs (human resource, medicines, health facilities, etc) compared to urban areas. It is very difficult to achieve equity in health financing and access with the NHIS if wider health system issues are not addressed as part of the design process. Equitable access will require improving availability of human resources and addressing disparities in distribution. Given the fact that there are districts where the only hospital is a PNFP hospital, there is urgent need to address HRH issues comprehensively to minimise attrition. Similarly, improving the level of functionality of the public health facilities, expansion of providers and effective regulation of and negotiations with the private sector needs to be addressed. The extent to which NHIS improves equity in access will depend on the level and distribution of resources by geographical regions and rural and urban areas. In addition, NHIS requires having structures and systems for its implementation that are currently not in place. This makes NHIS a relatively complex financing alternative, especially in Uganda where there is limited experience in managing NHIS systems.

Given that the informal sector joins the scheme in the seventh year, there is need to significantly increase the health sector allocation in order to improve services in the free wing where those who are not enrolled yet, including the poor who are largely in the informal sector, will continue to access services. Medium term expenditure framework projections for 2010/2011 to 2012/2013, shows no increase in MoH budget. There is also need for reorganisation and rearrangement of resource allocation especially at the hospital level. We recommend that the design of the NHI scheme should address wider health system issues as part of the preparation process. These include addressing human resource development, infrastructure development, and broader health financing issues (efficiency and equity in financing, integration of existing financing mechanisms, etc).

## Conclusion

Under the current design of the NHIS, only low coverage can be achieved initially. The important equity characteristics of pooling, cross-subsidisation and financial protection will not be optimally achieved with low coverage. Depending on the distribution of accredited providers, NHIS could worsen existing disparities in access to services, given the fee-for-service reimbursement mechanisms currently proposed as an option. Without a clear strategy for reducing fragmentation between existing health care financing systems, ensuring cross subsidisation, the NHIS will only serve to exacerbate fragmentation.

The government should use the introduction of NHIS as an opportunity to address several financing-related issues and to implement appropriate reforms. This means that NHIS introduction should not only be seen within the perspective of raising additional resources for the sector and of improving quality of health services, but also as a financing reform that appropriately addresses other important aspects of health care financing.

## Abbreviations

HC: Health centre; HI: Health insurance; HRH: Human resource for health; MOH: Ministry of health; NHIS: National health insurance scheme; NI: National insurance; OOP: Out of pocket; PNFP: Private not for profit; UGSHS: Uganda shillings; US$: United States dollars.

## Competing interests

The authors declare that they have no competing interests.

## Authors' contributions

JNO and CMZ contributed to the conceptualisation and drafting of the manuscript.

JN led the drafting process. Both authors read and approved the final manuscript.

## References

[B1] Braveman P Gruskin S Journal of epidemiology and community health 2003 57 254 258 10.1136/jech.57.4.254 12646539 PMC1732430

[B2] Van Doorslaer E Wagstaff A Rutten F editorsequity in the finance and delivery of health care, An international perspective 1993 New York: Oxford University press

[B3] WHO Health Financing: A strategy for the African Region 2006 AFR/RC56/10

[B4] Grant K Grant R Health insurance and the poor in low income countries 2003 World Hospital health services 39 1 19 22 12743884

[B5] Wagstaff A Van Doorslaer E Catastrophe and impoverishment in paying for health care with applications from Vietnam 1993 - 1998 Health Economics 2003 12 921 933 10.1002/hec.776 14601155

[B6] Wagstaff A Van Doorslaer E Culyer AJ, Newhouse JP Equity in Health Care Financing and Delivery; Chapter 40, North Holland Handbook of Health economics 1998

[B7] WHO Sustainable health financing, Universal coverage and social health insurance Geneva 2005 (A58/33)

[B8] Ministry of Health, Annual Health Sector Performance Reports for years; 2005/06 - 2008/09; Ministry of Health, Kampala, Uganda; 2006, 2009

[B9] MOH and Macro International Inc 2008 Uganda Service Provision Assessment Survey 2007. Ministry of Health and Macro International Inc.: Kampala, Uganda

[B10] Uganda Bureau of Statistics, Uganda National Household Survey 2005/2006: Report on the socio-economic survey. Uganda Bureau of Statistics: Kampala, Uganda 2006

[B11] Ministry of Health, Human Resources for Health Policy; Ministry of Health, Kampala, Uganda April 2006

[B12] Ministry of Health, Annual Health Sector Performance Report. Financial year 2007/08 Ministry of Health, Kampala, Uganda October 2008

[B13] Ministry of Health; Health facilities inventory, Ministry of Health: Kampala - Uganda, 2006

[B14] Ministry of Health; Report of the Mid-term review of the Health Sector Strategic Plan 2005/05 - 2009/10; Ministry of Health, Kampala, Uganda 2008

[B15] WHO World Health statistics 2010

[B16] Uganda Bureau of Statistics, National Service Delivery Survey; Uganda Bureau of Statistics, Kampala - Uganda; 2005

[B17] Ministry of Health, Annual Health Sector Performance Reports for Financial Years 2003/04, 2004/05, 2005/06, 2006/07, 2007/08; Ministry of Health, Kampala, Uganda; 2004, 2005, 2006, 2007, 2008

[B18] Ministry of Finance Planning and Economic Development; Background to the Budget 2008/09, Ministry of Finance Planning and Economic Development, Kampala, Uganda, 2009

[B19] Ministry of Health, Minimum Service Standards; Ministry of Health, Kampala, Uganda, 2008

[B20] World Bank, Ministry of Health, Ministry of Finance Planning and Economic Development; Fiscal space for health in Uganda; World Bank, Ministry of Health, Ministry of Finance Planning and Economic Development; Kampala, Uganda, 2010

[B21] Ministry of health; Annual Health sector performance review reports; 2008/09, Ministry of Health, Kampala, Uganda, 2009

[B22] Kutzin J A descriptive framework for country-level analysis of health care financing arrangements Health Policy 2001 56 3 171 204 http://www.ncbi.nlm.nih.gov/entrez/query.fcgi?cmd=Retrieve&db=PubMed&dopt=Citation&list_uids=11399345 10.1016/S0168-8510(00)00149-4 11399345

[B23] Government of Uganda; Constitution of the Government of Uganda; Kampala, Uganda; 1995

[B24] Ministry of Health; National Health Policy, Ministry of Health, Kampala, Uganda; 2000

[B25] Ministry of Health; Health Sector Strategic Plan 2000/01 - 2004/05 and 2005/06 - 2009/10, Ministry of Health, Kampala, Uganda, 2000, 2005

[B26] Berman Peter Hsiao William C Belli Paolo Bazeyo William Kasasa Simon Samuel Atuhurra A Feasibility Analysis of Social Health Insurance in Uganda, Kampala, Uganda 2001

